# Determination of Paraquat in Arabidopsis Tissues and Protoplasts by UHPLC-MS/MS

**DOI:** 10.21769/BioProtoc.4642

**Published:** 2023-04-05

**Authors:** Mingming Zhao, Qi Wang, Muyu Shi, Ziyan Sun, Huiru Tang, Xiaochun Ge

**Affiliations:** State Key Laboratory of Genetic Engineering, Department of Biochemistry and Biophysics, School of Life Sciences, Fudan University, Shanghai, 200438, China

**Keywords:** Paraquat, *Arabidopsis*, Oxidative stress, UHPLC-MS/MS

## Abstract

Paraquat is a cost-effective herbicide, widely used in many countries, that can induce severe oxidative stress in photosynthetic tissues. Studying plant herbicide resistance or antioxidant stress mechanisms requires determining the cellular paraquat level when plants are treated by paraquat. The traditional isotopic labeling method has the potential risk to cause problems to both human health and the environment. For radioisotope manipulation, special operation spaces and strict environmental inspection are also required. In addition, the radiolabeled paraquat is increasingly hard to buy due to the extended production cycle. Here, we describe a nonradioactive method to determine the paraquat level in a small number of *Arabidopsis* tissues or protoplasts, using a high resolution ultra-high-performance liquid chromatography (UHPLC)-mass spectrometry (MS)/MS method. This method is highly selective and sensitive, and more environmentally compatible and technically feasible than the isotope detection method.

## Background

Paraquat (PQ, 1,1-dimethyl-4,4-bipyridinium dichloride, or commonly called methyl viologen) is a nonselective herbicide that has been widely used in many countries for over half a century (Farrington et al., 1973; Fussell et al., 2011). It acts on chloroplasts and kills plants in several hours. PQ accepts electrons from photosystem I and transfers them to molecular oxygen, thus generating highly toxic reactive oxygen species (ROS) and causing severe oxidative stress in green tissues (Farrington et al., 1973; Fussell et al., 2011; Hawkes, 2014). In the past decades, a lot of PQ-resistant weeds have been found in the field. Studying plant resistance mechanisms to PQ not only helps to understand how weeds evolve herbicide resistance, but also promotes the understanding of plant antioxidant stress mechanisms. In *Arabidopsis*, a number of PQ-resistant mutants have been reported, including *rmv1 (resistant to methyl viologen 1*) (Fujita et al., 2012), *par1 (paraquat-resistant 1*) (Li et al., 2013), *atpdr11 (pleiotropic drug resistance 11*) (Xi et al., 2012), *rcd1 (radical-induced cell death 1*) (Fujibe et al., 2004), *pst1 (photoautotrophic salt tolerance 1*) (Tsugane et al., 1999), *par2 (paraquat-resistant 2*) (Chen et al., 2009), *pqt3 (paraquat tolerance 3*) (Luo et al., 2016), *dtx6* (Lv et al., 2021; Xia et al., 2021), and *atpqt11 (paraquat tolerance 11)* (Huang et al., 2022), and the mutant genes responsible for this resistance have been identified. The resistance mechanisms revealed so far have been classified into three types: impaired uptake and transport of PQ, enhanced sequestration of PQ, and enhanced ROS scavenging ability (Nazish et al., 2022).

We identified a new PQ-resistant mutant *rtp1 (resistant to paraquat 1)*, in which a MATE (multidrug and toxic compound extrusion) family protein DTX6 is disrupted (Lv et al., 2021). MATE family proteins are a type of transporters with distinct substrate specificity. To elucidate the resistance mechanism of DTX6, it is important to measure the PQ level in cells after treatment. The isotopic labeling method has been used to track exogenous molecules in plants; however, radiolabeled PQ is becoming increasingly difficult to obtain, and the manipulation of radioisotopes needs very complex approval and inspection procedures. Therefore, we established a nonradioactive method to determine PQ content in cells by combining ultra-high-performance liquid chromatography (UHPLC) and mass spectrometry (MS) systems. The UHPLC-MS/MS can determine PQ by using the multiple reaction monitoring mode of triple-quadrupole mass spectrometry. It has high selectivity and sensitivity and can achieve a resolution of 10^-15 ^mol/L on column. Compared with traditional radiolabeling, the UHPLC-MS/MS method is more environmentally compatible and easier to implement.

## Materials and Reagents

1. 50 mL conical centrifuge tube (PROMETHE, catalog number: PCT010500, sterile)
2. 15 mL conical centrifuge tube (PROMETHE, catalog number: PCT010150, sterile)
3. 2 mL centrifuge tube (Shanghai Jing Xin, catalog number: JX-LG0100, nonsterile)
4. 2.0 mL amber screw vial (Thermo Fisher, catalog number: C5000-2W)
5. 150 μL liner pipe (Thermo Fisher, catalog number: C4012-530)
6. Two-week-old *Arabidopsis* seedlings grown on 1/2 MS plates (mutant seeds are available via email request to the corresponding author, and wildtype seeds are available from TAIR website https://www.arabidopsis.org)
7. Four-week-old *Arabidopsis* plants grown in soil
8. Murashige & Skoog basal medium with vitamins (PhytoTech, catalog number: M519)
9. KCl (Sinopharm Chemical Reagent Co., Ltd, CAS: 7447-40-7)
10. CaCl_2_ (Solarbio, CAS: 10043-52-4)
11. NaCl (Sinopharm Chemical Reagent Co., Ltd, CAS: 7647-14-5)
12. HCl (Sinopharm Chemical Reagent Co., Ltd, CAS: 7647-01-0)
13. MES (BBI, CAS: 145224-94-8)
14. NH_4_OAC (Sinopharm Chemical Reagent Co., Ltd, CAS: 631-61-8)
15. Paraquat (Aladdin, CAS: 1910-42-5)
16. Water (LC-MS-grade) (Merck, Milli-Q IQ7000)
17. Methanol (LC-MS-grade) (Merck, CAS: 67-56-1)
18. Acetonitrile (LC-MS-grade) (Sigma-Aldrich, CAS: 75-05-8)
19. Formic acid (LC-MS-grade) (Sigma-Aldrich, CAS: 64-18-6)
20. Liquid nitrogen
21. W5 buffer (see Recipes)
22. Paraquat treatment buffer (see Recipes)
23. Wash buffer (see Recipes)
24. Paraquat extraction solution (see Recipes)
25. Redissolving solution (see Recipes)
26. LC mobile phase (see Recipes)

## Equipment

1. Automatic sample fast grinder (Shanghai Jing Xin, model: JXFSTPRP-CL)
2. Ultrasonic cleaners (Supmile, model: KQ-200VDE)
3. Nitrogen blowing concentrator (Beijing Jiayuan Industrial Technology Co., Ltd., model: MD200-2)
4. High-speed refrigerated centrifuge (KUBOTA, model: 6500)
5. Table high-speed centrifuge (Hettich, model: MIKRO 220R)
6. Light microscopy (Leica, model: Stereo microscope S8 APO)
-80 freezer
8. Water bath (HEDE Laboratory, model: DK-420BS)
9. Hemocytometer
10. UHPLC system (Shimadzu Nexera UHPLC LC30A system, including sample manager, solvent manager, and column oven)
11. Mass spectrometer system (SCIEX QTRAP 6500 plus mass spectrometer equipped with an electrospray ionization)
12. UHPLC column (ACQUITY UPLC BEH HILIC Column, 130Å, 1.7 µm particle size, 2.1 mm × 100 mm, Waters)

## Procedure

- **Treat *Arabidopsis* seedlings with paraquat**
  1. Surface-sterilize the *Arabidopsis* seeds with 70% ethanol and then grow them on vertical 1/2 Murashige & Skoog square plates (1% agar) for 10 days under standard long daylight growth conditions (16:8 h light/dark cycle) (Weigel and Glazebrook, 2002).
  2. Lay the plates horizontally and then leave them in darkness for two days to allow the hypocotyl to grow upward quickly. After that, return the plates to long daylight conditions again to grow for another two days (Figure 1).
    **Figure 1.**
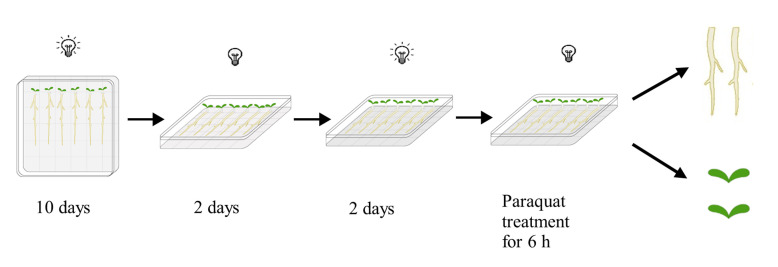
Brief diagram of seedling growth and treatment process
*Note: On each plate, mutants and the Col-0 wildtype control were grown side-by-side. The growth procedure illustrated in Figure 1 will facilitate the subsequent paraquat treatment and root harvest. Roots mainly grow on the surface of the media and can easily be treated and harvested, while leaves do not touch the media surface.*
  3. Use a pipette to add 10 mL of 10 μM paraquat slowly into the plates and let the paraquat distribute evenly on the media surface. Keep the plates horizontally in darkness for 6 h.
*Note: Use the same quantity of paraquat to treat each plate. The volume of paraquat should be enough to submerge the media surface but not enough to touch the leaves.*
  4. Harvest roots and leaves separately. Rinse the materials shortly three times with distilled water, blot them dry with paper towels, and then weigh. For mutants and controls, it is better to use a similar weight of materials for further experiments.
  5. Freeze the materials in liquid nitrogen and store them at -80 °C for paraquat determination.
- **Treat *Arabidopsis* protoplasts with paraquat**
  1. Prepare protoplasts from the leaves of *Arabidopsis* grown under short daylight conditions (8:16 h light/dark cycle) for four weeks based on the method described previously (Yoo et al., 2007). Suspend the protoplasts in cold W5 buffer.
  2. Count the protoplasts using a hemocytometer. Remove the W5 buffer and adjust the protoplasts to a final concentration of 2 × 10^6 ^protoplasts/mL with paraquat treatment solution. Use at least 10 mL protoplasts for one assay.
  3. Incubate the protoplasts in darkness at room temperature for 3 h. Gently shake the protoplasts by hand every 20 min.
*Note: This step is to load the paraquat into the protoplasts.*
  4. Centrifuge at 100 *× g* for 2 min at 4 °C and remove the supernatant carefully.
  5. Add 10 mL of wash buffer into the tube and resuspend the protoplasts gently.
  6. Repeat steps B4–B5 two more times.
*Note: The above steps can be used for paraquat uptake assay. The following steps are carried out for paraquat efflux assay.*
  7. After the final wash, suspend the protoplasts in wash buffer again and aliquot them equally into several tubes. Each tube corresponds to one time point. Set at least three replicates for each time point. For this assay, we use 0, 10, 30, and 60 min time points. Keep the tubes in darkness at room temperature and start timing using a timer.
  8. At each time point, centrifuge the tubes as in step B4. Collect the supernatants and protoplasts, respectively.
  9. Freeze the samples in liquid nitrogen and then store them at -80 °C for paraquat determination.
- **Paraquat extraction**
  1. Use 1 × 10^6^–2 × 10^6^ protoplasts or 20–30 mg of plant tissues to determine paraquat content in a 2 mL centrifuge tube. Conduct three freeze-thaw cycles (freeze the protoplasts in liquid nitrogen for 1 min, then thaw them in a water bath at room temperature for 2 min) to rupture the protoplasts. For plant tissues, grind the materials into powder in liquid nitrogen.
  2. Add 800 µL of paraquat extraction solution into each tube.
  3. Sonicate each sample for 20 min (1 min on, 1 min off) in total on ice.
  4. Put the samples in a water bath at 60 °C for 30 min and then cool down to room temperature.
  5. Centrifuge at 13,500 *× g* for 10 min at 4 °C and collect the supernatant.
  6. Repeat steps C2–C5 and combine the supernatants together.
  7. Evaporate the organic solvents with a nitrogen blowing concentrator and store the dried samples at -80 °C until determination.
- **Paraquat determination**
  1. Take the samples from -80 °C and add 100 μL of the room-temperature redissolving solution into the 2 mL centrifuge tubes to dissolve the samples.
  2. Lightly flick the tubes several times until the samples are completely dissolved. Centrifuge at 14,000 *× g* for 10 min at 4 °C. Pipette 80 μL of the supernatant into a 2 mL amber screw vial with a 150 μL liner pipe.
  3. Inject 1 μL of the sample into the system with a Waters BEH HILIC column (1.7 μm particle size, 2.1 mm × 100 mm).
  4. Perform a linear gradient elution program using LC mobile phase consisting of solvent A and solvent B. Set the flow rate of the mobile phase at 0.4 mL/min to conduct a linear gradient elution program (7 min): 90% B hold for 1 min, 90% B decreased to 20% B in 1 min, 20% B hold for 2.3 min, 20% B increased to 90% B in 0.2 min, and 90% B hold for 2.5 min. (The procedure was 0 min, 90% B; 1 min, 90% B; 2 min, 20% B; 4.3 min, 20% B; 4.5 min, 90% B; and 7 min, 90% B.)
  5. Detect the samples in positive mode (ESI+) with multiple reaction monitoring mode on the LC-MS/MS system.
    Set the gas parameters in ion source, including ion source gas 1, ion source gas 2, and the curtain gas to 55, 55, and 35 psi, respectively. Set the temperature to 500 °C and the ion spray voltage to 5,500 V. Monitor two multiple reaction transitions of each analyte for quantification and confirmation of paraquat (186.0–171.0 for quantification and 93.2–171.3 for confirmation). The voltages of declustering potential and collision energy are compound-specific parameters. For paraquat, they are 100, 60, 25, and 17, respectively. Set other parameters to default values as recommended by the manufacturer.
  6. Integrate the chromatographic peak of the target analyte. Integration parameters were set to 9 and 1,000 points for the minimum peak width and height, and S/N integration threshold and baseline subtraction window were set to 3 and 2 min, respectively.

## Data analysis

1. Calculate the chromatographic peak area of paraquat directly by UHPLC-MS/MS. The peak area of paraquat represents the amount of analyte on the column. Since the injection volume is 1 µL and the total volume of the sample is 100 µL, we multiply the chromatographic peak area by 100 to obtain the total quantity of analyte in the sample.
2. Paraquat efflux assay results are presented in Figure 2A. The same number of protoplasts was sampled at each time point for paraquat determination. Paraquat content in protoplasts was indicated by its chromatographic peak area. The paraquat level at time point 0 was set as 100% and the relative levels of paraquat at other time points were calculated according to the formula in Figure 2B.
3. Root uptake assay results are presented in Figure 2C. The normalized paraquat level in tissues was obtained by dividing the chromatographic peak area of paraquat by the quantity of materials, that is, the weight of *Arabidopsis* materials (such as roots and leaves). The paraquat content in different samples was compared.
  **Figure 2.**
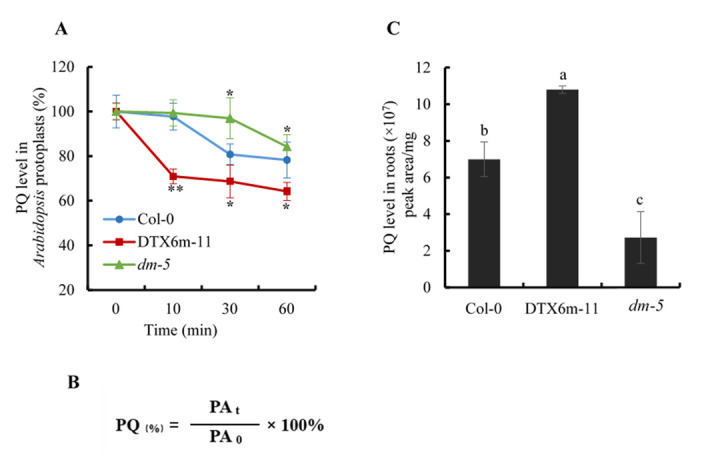
Paraquat determination in protoplasts and plant tissues by UHPLC-MS/MS. (A) Paraquat efflux assay using *Arabidopsis* leaf protoplasts. The paraquat level in protoplasts was indicated by its chromatographic peak area. At least three replicates for each time point were determined. The paraquat level at time point 0 was set as 100%, and the relative levels of paraquat at other time points were calculated using the formula given in Figure 2(B). The samples shown are Col-0 (wildtype control), DTX6m-11 (DTX6m-overexpression line), and *dm-5 (dtx5 dtx6* double mutant) (Lv et al., 2021). Values are mean ± SD (n = 3). Significance of the differences between Col-0 and the other materials is analyzed by Student’s t-test. PQ, paraquat. ** *P* ≤ 0.01; * *P* ≤ 0.05. (B) Calculation formula of relative paraquat level at different time points in *Arabidopsis* protoplasts. PA_t_: peak area of paraquat at each time point; PA_0_: peak area of paraquat at time point 0. (C) Paraquat uptake assay using *Arabidopsis* roots. *Arabidopsis* were treated with 10 μM paraquat in darkness for 6 h and the paraquat level in tissues was defined as the chromatographic peak area of paraquat divided by the fresh weight of the tissues. Values are mean ± SD (n = 3). Two-way ANOVA is performed to show the significance of difference.

## Recipes

1. **W5 buffer (for protoplast experiment)**
  154 mM NaCl
  125 mM CaCl_2_
  5 mM KCl
  2 mM MES (pH 5.7)
*Note: Prepare mother solutions for each reagent, then filter and store them at 4 °C. Make W5 buffer freshly using mother solutions.*
2. **Paraquat treatment solution (for protoplast experiment)**
  154 mM NaCl
  125 mM CaCl_2_
  5 mM KCl
  2 mM MES (pH 5.7)
  5 mM paraquat
*Note: Make this solution freshly using the stored mother solutions of each component. The mother solution of paraquat should be stored at -4 °C in the dark.*
3. **Wash buffer (for protoplast experiment)**
  154 mM NaCl
  125 mM CaCl_2_
  5 mM KCl
  2 mM MES (pH 5.7)
*Note: Make wash buffer freshly using the stored mother solutions.*
4. **Paraquat extraction solution**
  60% methanol
  40% 0.5 M HCl
*Note: This solution can be stored at -20 °C for three months.*
5. **Redissolving solution**
  50% acetonitrile
  50% Milli-Q water
*Note: Make this solution freshly.*
6. **LC mobile phase**
  Solvent A (pH = 3.95):
  100 mM NH_4_OAC
  0.5% formic acid in water
  Solvent B:
  Acetonitrile
*Note: Make solvent A freshly.*
